# Creation of an international laboratory network towards global microplastics monitoring harmonisation

**DOI:** 10.1038/s41598-024-62176-y

**Published:** 2024-06-03

**Authors:** Adil Bakir, Alexandra R. McGoran, Briony Silburn, Josie Russell, Holly Nel, Amy L. Lusher, Ruth Amos, Ronick S. Shadrack, Shareen J. Arnold, Cecy Castillo, Joaquin F. Urbina, Eduardo Barrientos, Henry Sanchez, Keshnee Pillay, Lucienne Human, Tarryn Swartbooi, Muhammad Reza Cordova, Sofia Yuniar Sani, T. W. A. Wasantha Wijesinghe, A. A. Deeptha Amarathunga, Jagath Gunasekara, Sudarshana Somasiri, Kushani Mahatantila, Sureka Liyanage, Moritz Müller, Yet Yin Hee, Deo Florence Onda, Khairiatul Mardiana Jansar, Zana Shiraz, Hana Amir, Andrew G. Mayes

**Affiliations:** 1 Centre for Environment, Fisheries and Aquaculture Science (Cefas), Pakefield Road, Lowestoft, Suffolk, NR33 0HT UK; 2 Norwegian Institute for Water Research (NIVA), 0579 Oslo, Norway; 3 Vanuatu Bureau of Standards, PO Box 6532, Port Vila, Vanuatu; 4 Faculty of Science and Technology, University of Belize, Hummingbird Avenue, Belmopan City, Belize; 5 Department of Marine Biosciences, Tokyo University of Marine Science and Technology, 4 5-7, Konan, Minato-Ku, Tokyo, 108-8477 Japan; 6 Department of Environment, 7552 Hummingbird Hwy, Belmopan City, Belize; 7 Department of Forestry, Fisheries and the Environment (DEFF), 2Nd Floor Foretrust Building, Martin Hammerschlag Way, Foreshore, Cape Town, 8001 South Africa; 8 South African Environmental Observation Network (SAEON), Nelson Mandela University Ocean Sciences Campus, 4 Gomery Avenue, Summerstrand, Port Elizabeth, South Africa; 9 Botany Department, Institute of Coastal and Marine Research (CMR), Nelson Mandela University, Summerstrand South Campus, PO Box 77 000, Port Elizabeth, 6031 South Africa; 10 Research Center for Oceanography, The Indonesian National Research and Innovation Agency (BRIN), Kawasan Jakarta Ancol Jl Pasir Putih 1 Ancol, Jakarta, 14430 Indonesia; 11 Central Environmental Authority, No 104, Parisara Piyasa, Densil Kobbekaduwa Mawatha, Battaramulla, Sri Lanka; 12 Environmental Studies Division, National Aquatic Resource Research & Development Agency, Crow Island, Colombo, 15 Sri Lanka; 13 Marine Environment Protection Authority, No.177, Nawala Road, Narahenpita, Colombo, 05 Sri Lanka; 14 Industrial Technology Institute, No. 363, Bauddhaloka Mawatha, Colombo, 07 Sri Lanka; 15 Faculty of Engineering, Computing and Science, Swinburne University of Technology Sarawak Campus, Jalan Simpang Tiga, 93350 Kuching, Sarawak Malaysia; 16 Institute of Oceanography and Environment, Universiti Malaysia Terengganu, 21300 Kuala Nerus, Terengganu Malaysia; 17 Microbial Oceanography Laboratory, The Marine Science Institute, University of the Philippines, Diliman Quezon City, Philippines; 18 Department of Earth Science and Environment, Faculty of Science and Technology, Universiti Kebangsaan Malaysia, 43600 Bangi, Selangor Malaysia; 19 School of Chemistry, University of East Anglia, Norwich Research Park, Norwich, NR4 7TJ UK; 20 Maldives Marine Research Institute, Ministry of Fisheries and Ocean Resources, H. White waves, Moonlight Hingun Magu, Male′, 20025 Maldives

**Keywords:** International network, Microplastics, Global plastics treaty, Nile red, Plastic pollution, Environmental sciences, Ocean sciences

## Abstract

Infrastructure is often a limiting factor in microplastics research impacting the production of scientific outputs and monitoring data. International projects are therefore required to promote collaboration and development of national and regional scientific hubs. The Commonwealth Litter Programme and the Ocean Country Partnership Programme were developed to support Global South countries to take actions on plastics entering the oceans. An international laboratory network was developed to provide the infrastructure and in country capacity to conduct the collection and processing of microplastics in environmental samples. The laboratory network was also extended to include a network developed by the University of East Anglia, UK. All the laboratories were provided with similar equipment for the collection, processing and analysis of microplastics in environmental samples. Harmonised protocols and training were also provided in country during laboratory setup to ensure comparability of quality-controlled outputs between laboratories. Such large networks are needed to produce comparable baseline and monitoring assessments.

## Introduction

Global solutions to reduce plastic pollution reside in global collaboration. Plastic pollution has been reported for all environmental compartments globally and has gained significant public awareness over the past years due to its widespread distribution and adverse ecological and economic effects attracting widespread publicity. Plastics can be present at different size scales from large items (e.g. mega (> 1 m)- and macroplastics (1 m to 2.5 cm) to smaller particles from meso- (5 mm to 2.5 cm), to micro- (below 5 mm in size) and nanoplastics (1–1000 nm)^1,2^. Microplastics (MPs) have been detected in all environmental compartments investigated. Their ubiquity has led to increased concerns about their potential adverse environmental impacts. They have previously been shown to be ingested by terrestrial, freshwater and marine organisms with some evidence of bioaccumulation in tissues^3^. They have also been shown to enter the food chain^4^ from airborne vectors^5^ as well from the combined pathways through food, water and air^6^. Results from correlative studies in people exposed to high concentrations of microplastics, and model animal and cell culture experiments, suggest that effects of microplastics could include provoking immune and stress responses and inducing reproductive and developmental toxicity^7^.

The above-mentioned concerns about microplastics have highlighted the need to understand their current spatial distribution as well as temporal changes, which relies on monitoring programmes. To understand global changes, it requires, first, the knowledge of local, national and regional scale variations before action at an international level can be achieved. To measure this, initial baseline data are required. Following this, measurable parameters can be implemented to quantify changes following remediation measures or to assess the effectiveness of regulatory actions. While the field of microplastics has seen an explosion in scientific outputs over the last years, many knowledge gaps still need to be addressed, especially related to monitoring data for some areas. Due to the relative newness of the field of microplastic research, spatial data is patchy at a global scale and very little reliable temporal data is available since baseline values for comparison do not yet exist in most areas.

Unlike traditional environmental pollutants that have well established and standardised methods for their detection and quantification (i.e. metals, hydrophobic organic compounds), microplastics are diverse, consisting of many different polymer and associated chemical combinations^8^. This situation has led to a suite of methodologies (at varying Technological Readiness Levels) and no single standardised approach, with variations occurring at every step of the analytical chain and limited understanding of the consequence of the variation introduced with modifications to fundamental steps, making direct comparisons between datasets difficult^9^. Therefore, a large-scale, reliable international coordination of reporting and monitoring programmes for microplastics is needed. This is most relevant at a time where there is movement towards a global agreement on plastic pollution (UNEA-5.2) and to support Sustainable Development Goal (14), which specifically relates to reducing impacts from plastics, including microplastics (indicator 14.1.1 b). There are efforts from The Joint Group of Experts of Marine Environmental Protection (GESAMP) and also regional efforts where there are some regulatory requirements (Marine Strategy Framework Directive, OSPAR Convention and Arctic Council) to work towards ways of reporting on microplastics and produce guidelines to better characterise microplastic pollution. Therefore, there is a need for more focus on standardisation, to enable comparison and merging of data from research across the world, in order to be able to provide reliable assessments to tackle this global problem. Such monitoring programmes should include common research themes covering the installation of common infrastructure with the use of harmonised guidelines allowing for the development of comparable scientific outputs. Examples of such large-scale international projects include the Commonwealth Litter Programme (CLiP) and the Ocean Country Partnership Programme (OCPP) as described below.

### The Commonwealth Litter Programme (CLiP) and the Ocean Country Partnership Programme (OCPP)

**Figure 1:**
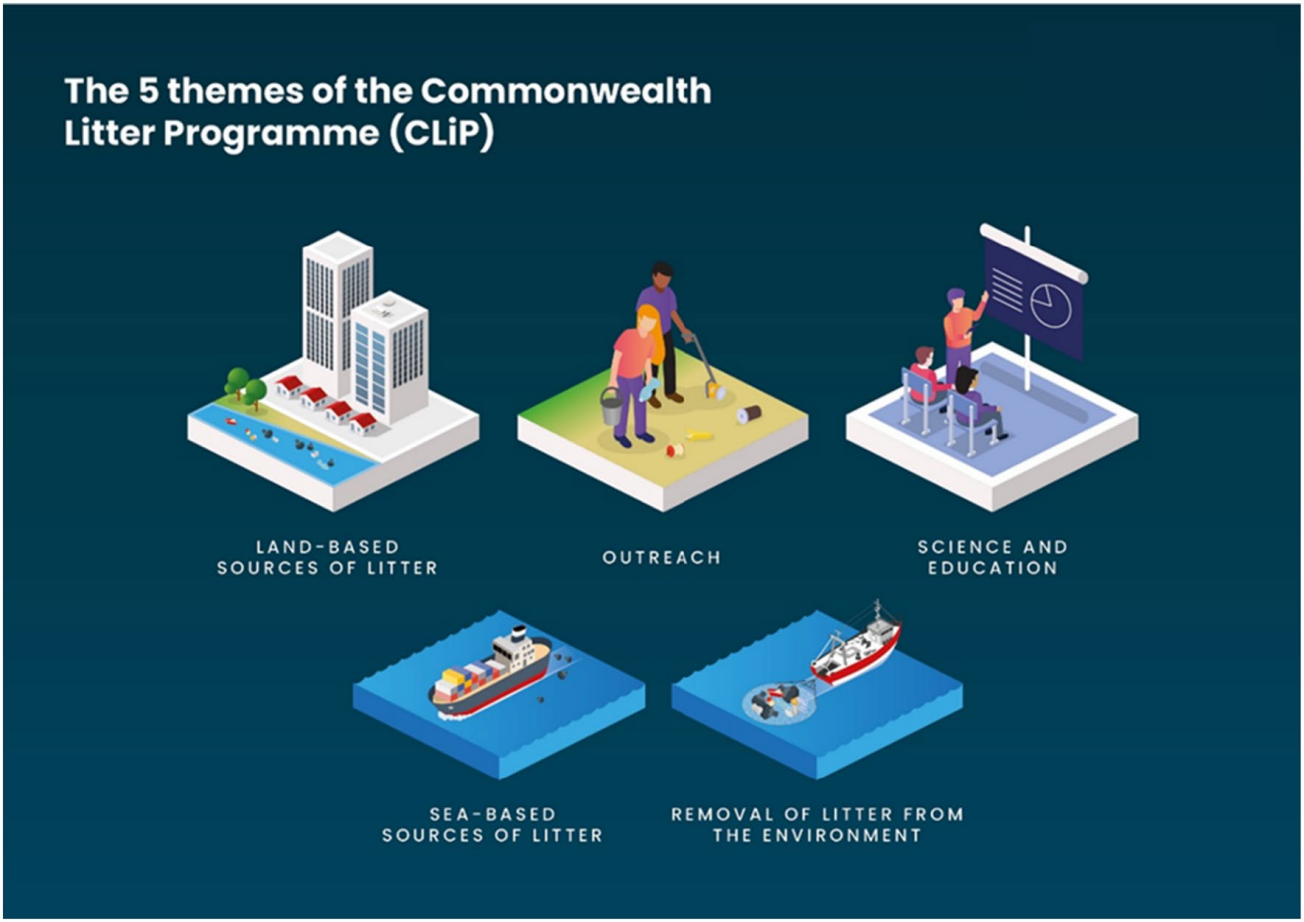
Five pillars covered by the Commonwealth Litter Programme (CLiP) (2018–2021)^11^.

CLiP has been funded by the Government of the United Kingdom and implemented by the Centre for Environment, Fisheries and Aquaculture Science (Cefas). Under the Commonwealth Blue Charter, the United Kingdom and Vanuatu are leading the Commonwealth Clean Ocean Alliance (CCOA), an action group for tackling marine plastic pollution. CLiP was one of several programmes to help countries meet their responsibilities under CCOA. The overall goal of CLiP was to reduce the amount of marine litter which ends up in the world’s ocean and ameliorate the negative impact of marine litter on economies, communities and livelihoods, particularly in key industries such as fisheries and tourism. The programme impact contributed to this by assisting selected Commonwealth countries to adopt national Marine Litter Action Plans (MLAPs) with a view to implementation^10^. To get to this point, the programme aimed to improve national capacities to tackle marine litter, initiate long-term behavioural change by helping communities better understand the problem of marine litter, and support the development and implementation of national MLAPs. CLiP was operated according to five pillars, namely (i) Land-based sources of litter, (ii) Sea-based sources of litter, (iii) Removal of litter from the marine environment, (iv) Science and Education and (v) Outreach (Fig. 1). The project was completed in 2021 and was operated in Vanuatu, Solomon Islands, Belize, South Africa (SA), Sri Lanka and India.

Upon completion of CLiP, OCPP was launched by the UK Government under the Blue Planet Fund (BPF). This -Official Development Assistance (ODA) programme funded by the UK’s Department for Environment, Food and Rural Affairs (Defra) also makes funding contributions to the Global Ocean Accounts Partnership (GOAP) and Friends of Ocean Action (FOA). GOAP partners with ODA-eligible countries to calculate and account for the value that a healthy ocean brings to their economies, by supporting them to develop ocean natural capital accounts. FOA is hosted by the World Economic Forum in collaboration with the World Resources Institute, which brings together ocean leaders from a wide range of sectors to encourage action and investment into sustainable ocean projects. Through the OCPP, Cefas, in partnership with JNCC and MMO, provide technical assistance to support countries to tackle marine pollution, support sustainable seafood practices and establish designated, well-managed and enforced Marine Protected Areas (MPAs). Cefas is currently leading the delivery of the ‘Marine Pollution’ and ‘Sustainable Seafood’ themes, working in partnership with experts from JNCC and MMO. The ‘Marine Pollution’ strand built on work carried out during CLiP in Vanuatu, Solomon Islands, Belize, SA, Sri Lanka and India, as well as forging new relationships in the Maldives, Mozambique, Senegal and Ghana. Under the ‘Science and Education’ pillar of CLiP (and consequently OCPP) several laboratories were created in SA, Belize, Sri Lanka, the Maldives and in the South Pacific in collaboration with project partners. The main aim of the newly created facilities was to provide the adequate infrastructure and harmonised protocols to develop and carry out monitoring programmes for the production of data as scientific evidence in support of regulatory actions and national or regional MLAPs.

In parallel with the Cefas activities, UEA initiated a similar programme of capacity building and training in microplastic analysis for Malaysia, funded by the UK government through the Global Challenges Research Programme (https://www.uea.ac.uk/groups-and-centres/global-research-translation-award-project/microplastics). The project had a slightly different approach, since it supported existing academic laboratories with experience of environmental sampling and analysis, but which lacked the specialised equipment and expertise. The aim was to provide equipment and training, thus building capacity and expertise to raise awareness of the issues of microplastics and facilitate a variety of assessment and monitoring programmes. This was assisted by forming the Malaysian Microplastics Network (MyMiP—https://mmp.umt.edu.my). Since UEA and Cefas have worked closely on the development of analytical approaches for rapid monitoring of microplastics based on fluorescence staining with Nile Red, similar equipment and training was provided to this network, so capabilities and opportunities to network and share outputs are very comparable.

Other international organisations are also aiming at harmonising collection protocols for the sampling, separation and identification of microplastics in environmental samples. The International Atomic Energy Agency (IAEA), within its NUclear TEChnology for Controlling Plastic Pollution (NUTEC Plastics—https://www.iaea.org/services/key-programmes/nutec-plastics) initiative is supporting countries in the Western Pacific rim to develop harmonised protocols to produce robust marine plastic pollution data. Collaboration between UEA, MyMiP and Kyushu University (Japan) also created an opportunity to extend this provision of protocols, training and imaging equipment to the Philippines, and also to Thailand in the near future as part of a wider long-term vision to support plastic pollution assessment throught the ASEAN region. However, the development of global harmonised protocols, and in some instances infrastructure, need to be considered alongside sustainable financing to support maintenance of equipment, restocking of consumables and employment of laboratory personnel if such initiatives are to have long-term impact.

This document provides a guide for the creation of a cost-effective microplastic laboratory in areas for which a lack of infrastructure is a major barrier in the production of scientific outputs in the field of microplastics research. Additionally, by sharing ambitions and lessons learnt from individual microplastics analytical laboratories involved in CLiP, OCPP and MyMIP, we hope to facilitate inter-lab connections and increase visibility to allow for a better connectivity between national and regional bodies working towards the same goals. The main objectives are to: i) review and present an already existing international microplastics laboratory network, ii) to provide an overview of the type of infrastructure and training required to develop such laboratories, iii) to present a list of current and planned activities for the different laboratories and to initiate connectivity and dialogue between the different areas on a national, regional and international level, and iv) to propose one of the first interlaboratory proficiency test focusing on the use of Nile Red (NR) coupled with FTIR for the analysis of microplastics in environmental samples.

### Current laboratory network

**Figure 2:**
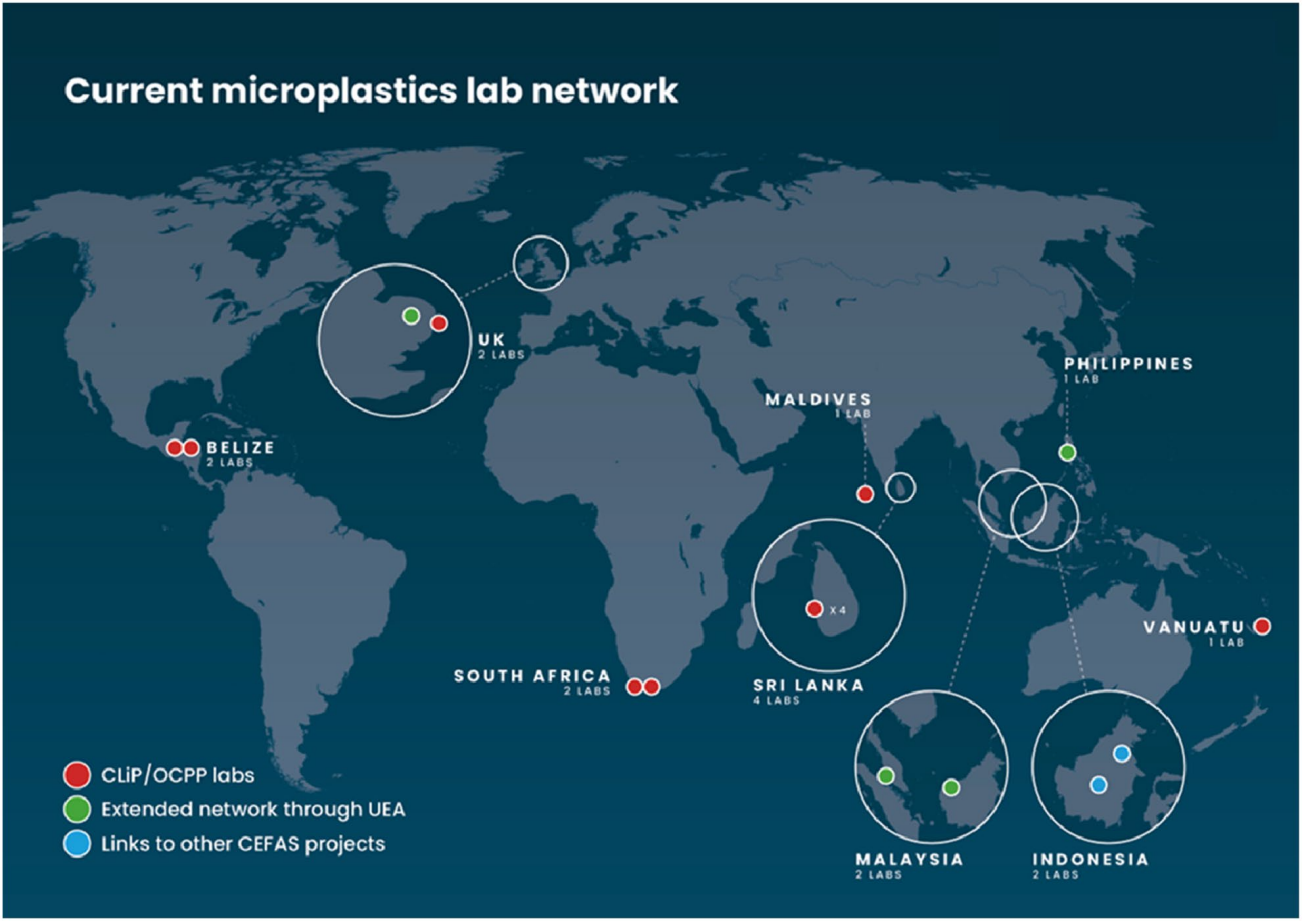
Current microplastics laboratory network.

The current laboratory network is composed of facilities (n = 15) in Vanuatu, Belize, South Africa, Sri Lanka, the Maldives, Indonesia, the Philippines and Malaysia (Fig. 2). The network is mainly composed of governmental bodies and universities in Europe, Central America and the Caribbean, Africa, Asia and the South Pacific.

### Low-cost and rapid screening of microplastics in environmental samples using fluorescence tagging of polymers with Nile red (NR)

Microplastics have been found on beaches, coastal zones, open-sea and deep-sea sediments globally. Microplastics monitoring data are widely accessible for some environmental compartments for various locations worldwide^12^. The lack of implemented harmonised monitoring guidelines is, however, making comparison between datasets difficult^13^. As a result, effective monitoring in the environment is of global concern and urgently needs development of common indicators for different environmental compartments^14^. There is also a significant gap in monitoring data for some geographically remote areas for which scientific infrastructures are lacking. Difficulties in accessing appropriate infrastructure has been defined as a significant barrier for the production of scientific outputs and monitoring data required for the development of a holistic understanding of microplastics^15^. Microplastic research often requires costly and technically advanced equipment (Table 1) not widely affordable. Difficulties in sourcing costly chemicals directly in country due to cost or from an absence of local suppliers is also a limiting factor. Instead, there is a need for low-cost analytical techniques for the large-scale mapping of microplastics. NR was developed as a low-cost and rapid approach for the detection and quantification of microplastics in environmental samples by the University of East Anglia (UEA) and Cefas^16^. Since its development, the application of NR in relation to microplastic research has increased substantially^17^. Shruti et al. (2021) recently published a review on the application of NR for the analysis of microplastics in environmental samples including food products. While the need for standardised protocols for NR use was highlighted in the review and by others^17–25^, the authors concluded that NR tagging of microplastics was a promising approach for a cost effective and rapid screening of microplastics from environmental samples, especially for laboratories lacking more advanced and often costly infrastructure (e.g. pyrolysis GC–MS or μ-FTIR, μ-Raman facilities). NR has also previously been used for the large-scale mapping of microplastics from sediment, indicating its suitability in a monitoring context^14,26,27^ and has also been applied to the detection and quantification of microplastics in biota^28–31^ and water^17,26,30–32^ NR has also been applied for freshwater, estuarine and marine studies to understand microplastics fluxes from sources to sinks^33–35^. Despite recent advances in NR staining techniques for microplastics detection, further developments and standardisation are required to establish NR as an efficient standalone method for environmental monitoring of microplastics. Some limitations still have to be addressed including the effective staining of some coloured and black plastics with NR, interference from naturally fluorescent particles and associated organic matter and standardisation of imaging systems including lighting conditions, including wavelength and intensity^19^.

**Table 1:** Microplastics particle identification technical overview ranked by cost (Updated from Thermoscientific (2020) and Bakir et al. 2020^36,37^.

Analytical method	Minimum particle size (μm)	Filter requirement	Degree of automation	Acquisition speed	Advantages	Disadvantages	Relative cost
ATR-FTIR spectroscopy	> 500 (smaller with care)	N/A	Very low	Fast	- Ease of use - Minimum sample preparation	Contact analysis (ATR)	$
FTIR microscopy	> 10	IR transparent (transmittance measurement configuration)	Low to High (microscope dependent)	Fast	- Ease of use - Minimum sample preparation-		$$
ATR-FTIR spectroscopy with microscope attached	> 5	Any filter Any substrate	High	Medium	- Ease of use - Minimum sample preparation	Contact analysis (ATR)	$$
FTIR imaging	> 5	IR transparent (transmittance measurement configuration)	Very high	Very fast	- Ease of use - Minimum sample preparation		$$$
ATR-FTIR imaging	> 2	Any filter Any substrate	High	Medium	- Ease of use - Minimum sample preparation	Contact analysis (ATR)	$$$
LDIR imaging	> 10	Flat, reflective surface (e.g. Kevley slide or IR reflective filter such as gold filters)	High	Very fast		Less commonly used than FTIR, limited reference spectra. Requires more validation for environmental samples	$$$
Raman imaging	> 0.5	Non-fluorescent	Very high	Fast	Resolving particles down to 1 micron and less	Less commonly used than FTIR, limited reference spectra	$$$
Thermal analysis		N/A			- Suitable for nanoplastics identification - Analysis of polymer type and additive chemicals	- Destructive analysis - Reporting unit (mass vs number) - Complex data (pyr-GC–MS	$$$

## The need for harmonised guidelines and reporting

**Figure 3:**
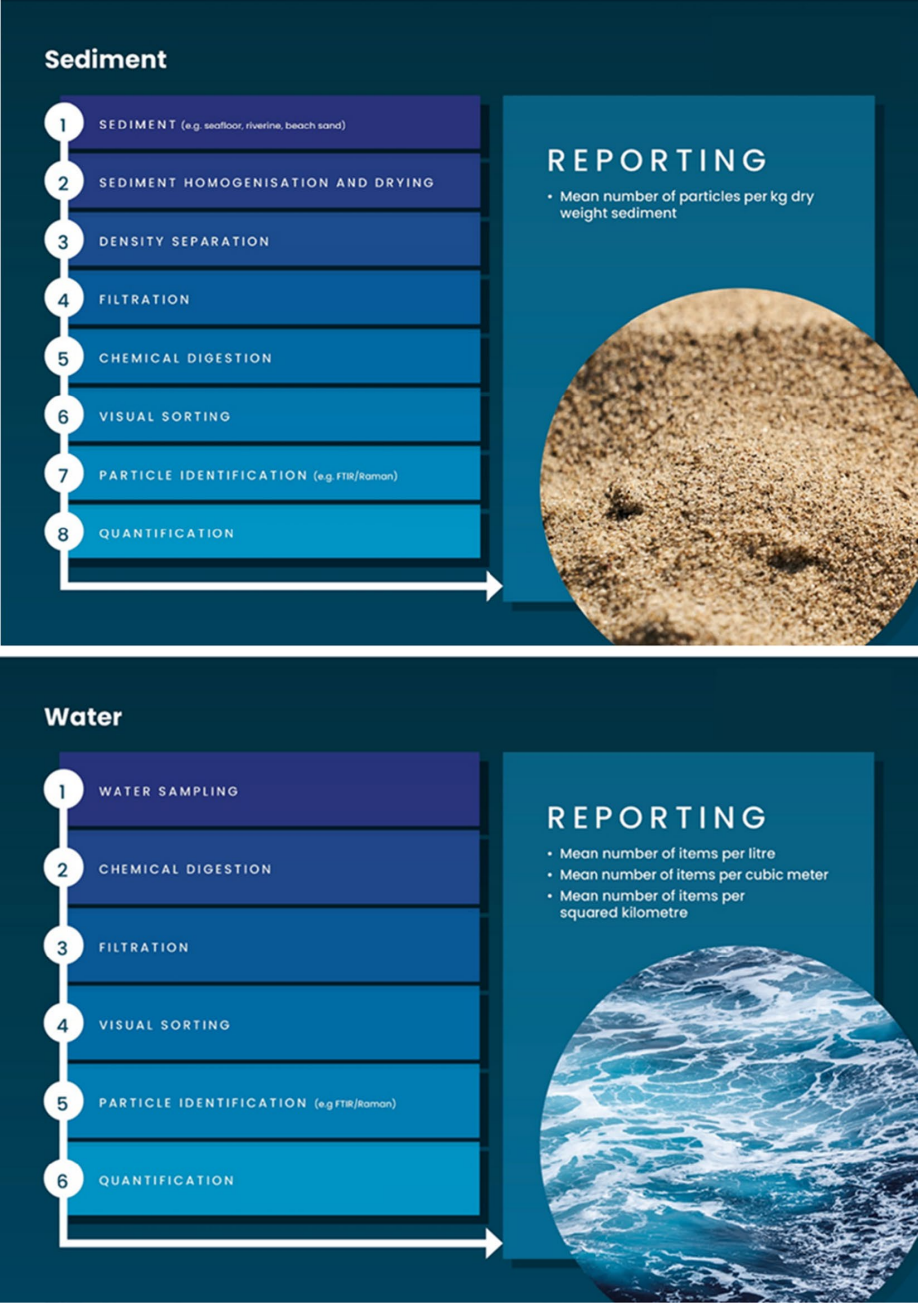

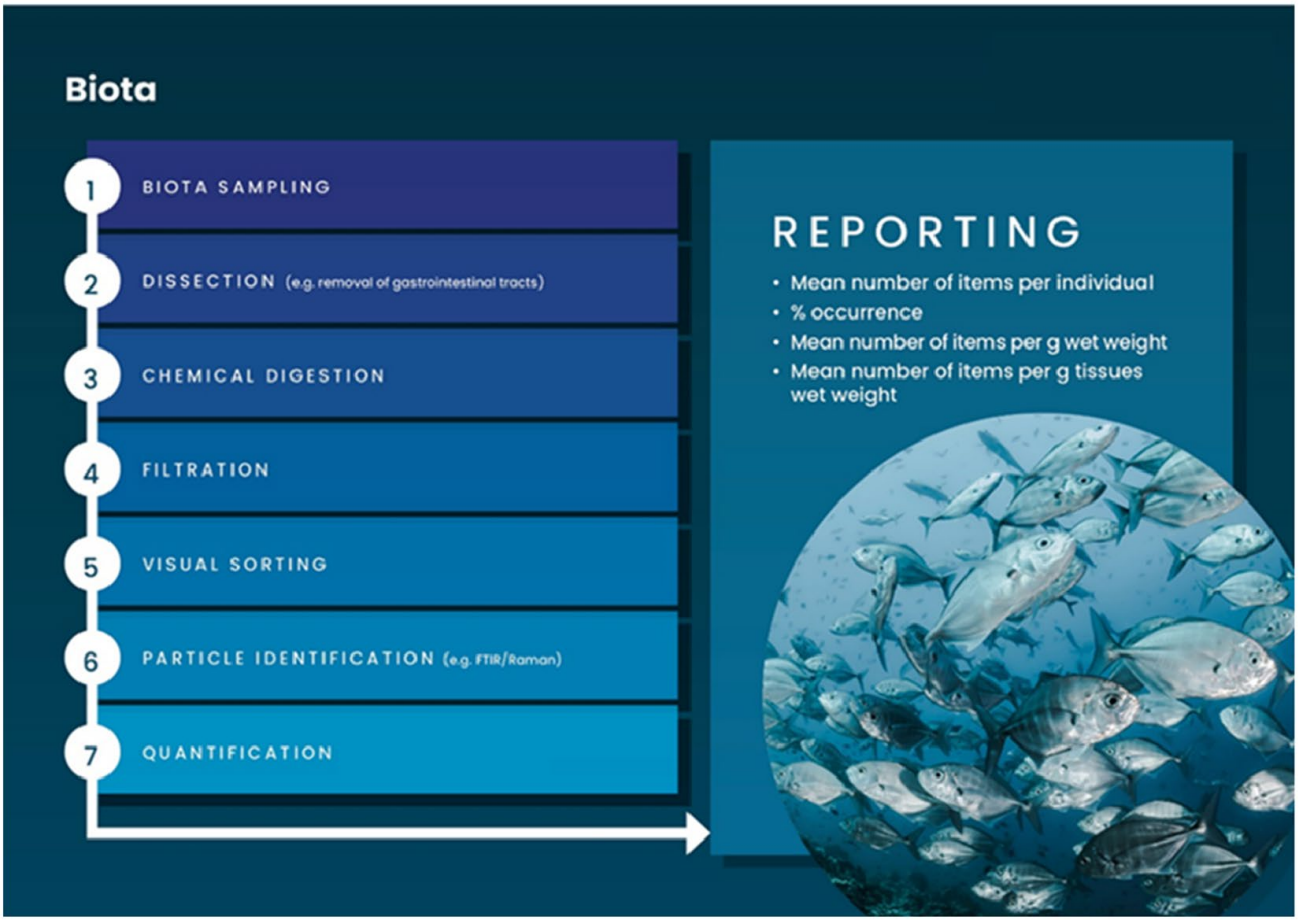
Flow charts for the sampling, extraction, isolation, analysis and reporting of microplastics for environmental samples (i.e. sediment, water and biota).

The need for the use of harmonised protocols for the sampling, processing and reporting of microplastics in environmental data has been voiced by many studies over the past years^9,38^. Generally, individual processes for the extraction, isolation, identification and quantification of microplastics in environmental samples are already harmonised and are presented in Fig. 3 for sediment, water and biota. SOPs were developed according to previously published international guidelines^2,39–44^. Best practices, identified from international interlaboratory proficiency tests or method development exercises (Table 4), were also implemented in the different protocols^45^. Harmonised protocols should also be flexible and account for local context and requirements which may vary regionally and globally. Each SOP was further optimised and validated in country before adoption and application. Different matrices require different levels of sample preparation with contrasting levels of complexity (Fig. 3). The different laboratories ranked the different environmental compartments with an increasing level of complexity as follows: atmospheric, water, beach sand, biota and sediment, identified as the main problematic matrix.

## The need for infrastructure

As described previously, access to suitable scientific infrastructure is a limiting factor in the production of scientific outputs. One solution is collaboration. Collaborative work can be translated in several ways and could be through international schemes as well as regional or national cross-institutional partnerships. One of the main focuses of CliP, OCPP and MyMiP was to provide adequate national facilities to support scientific advances in the field of microplastics.

The main criteria to ensure rapid implementation of ready-to-use facilities were as follows:

(i) Secure some working space with local partners. The current laboratories were based on existing laboratory facilities.
(ii) Acquisition of cost-effective equipment and low-risk chemicals.
(iii) Use local expertise to assess method limitations and to optimise Standard Operation Procedures (SOPs).

**Figure 4:**
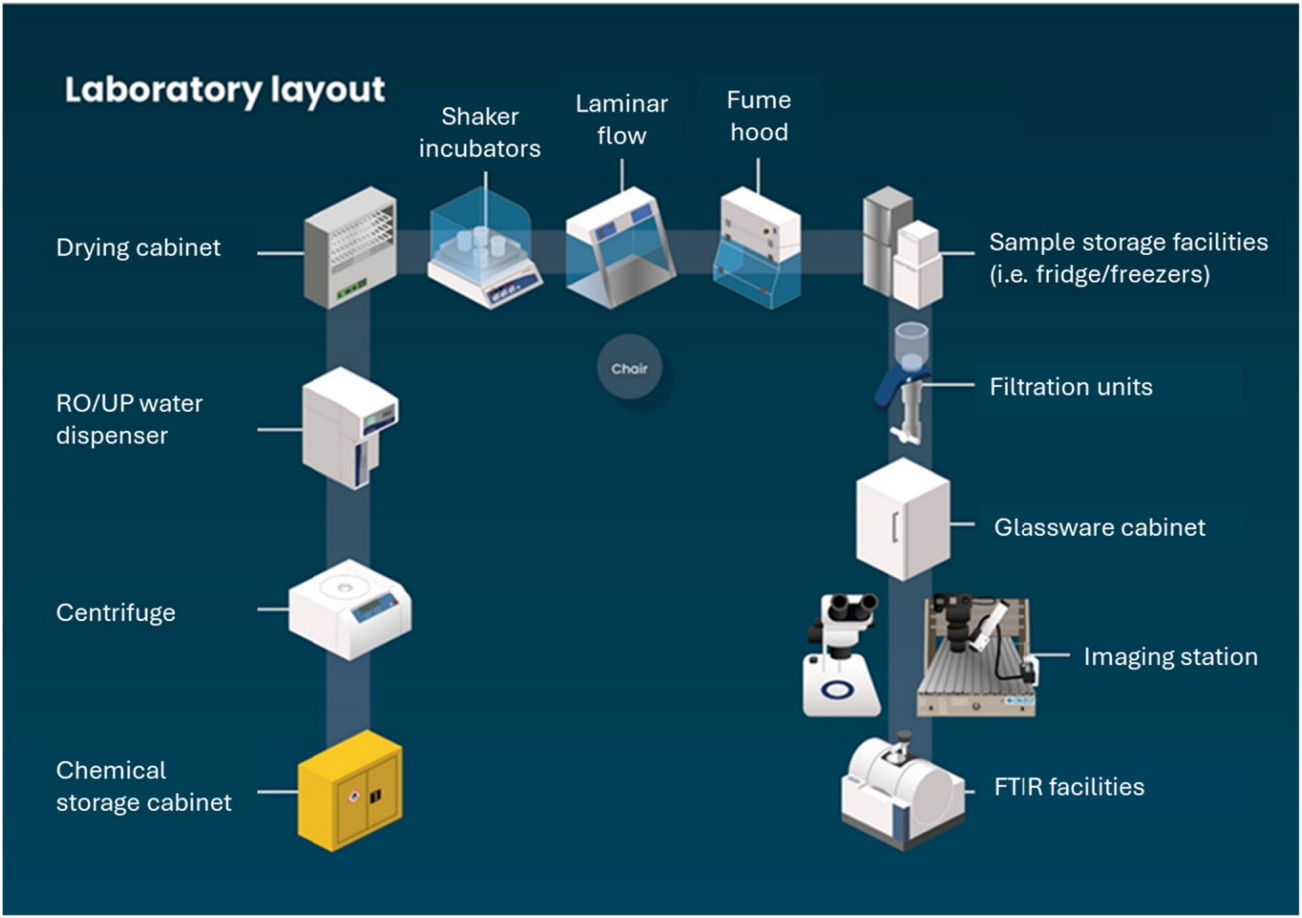
Current layout of the laboratories for the CLiP/OCPP laboratories as well as for the laboratories created via other Cefas projects.

Laboratory items can be classified into ‘must have’, ‘should have’, ‘could have’ and ‘won’t have’ (MoSCoW). By using the MoSCoW prioritisation method, equipment needs can be determined according to the environmental compartment to be targeted (Table 2). Subsequently, laboratories are equipped with key items to allow for the sampling, processing and analysis of microplastics in environmental samples (Fig. 3). The list of equipment was mainly based on the current Cefas microplastics laboratory setup (Fig. 4). General processes and the choice of chemicals were determined from requirements identified from SOPs (Fig. 3) as well as experts’ opinions. Special attention was given to minimise background contamination during sample handling and processing of environmental samples with the use of laminar flows to avoid dust contamination as well as filtration units and filters to remove fine plastic particles from chemicals and reagents (Table 2)^46^. Key items including shaker incubators and chemicals (e.g. KOH) for sample digestion and removal of biogenic matter were also provided^47^. The list of equipment was presented to each laboratory for validation before shipping.

**Table 2:**
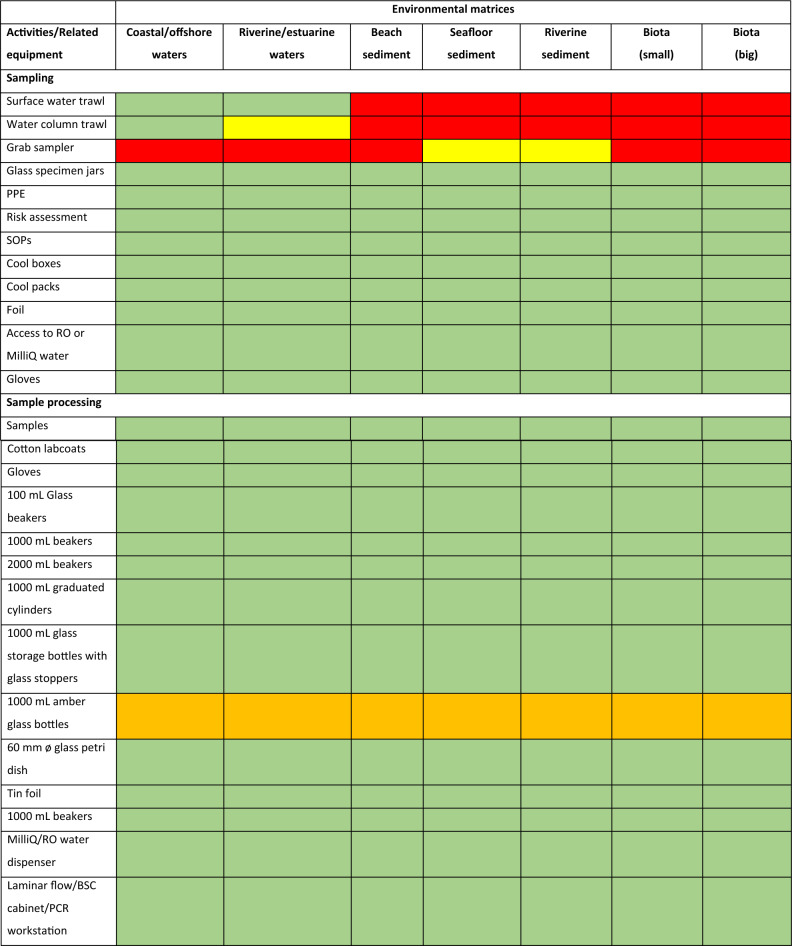

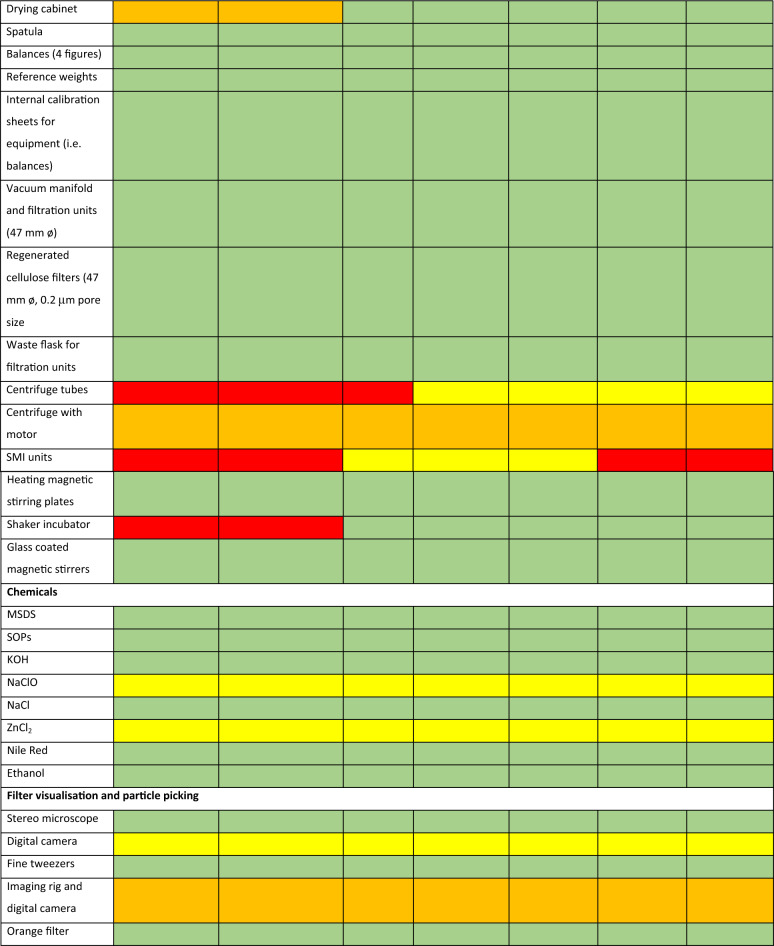

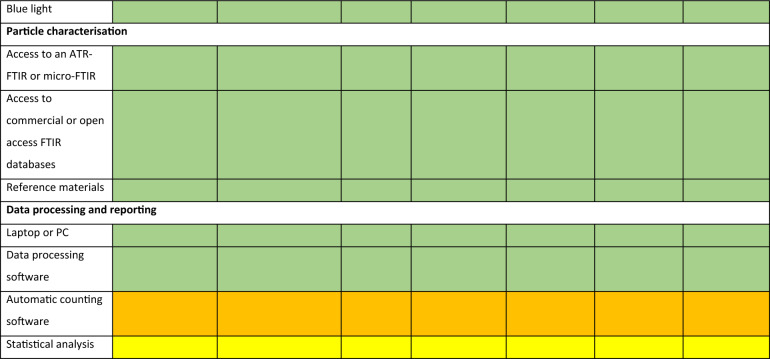
List of equipment required for the creation of a microplastics laboratory based on the use of Nile red and using the MoSCoW prioritisation method. M-Must have (green), S-Should have (yellow), C-Could have (orange) and W-Won’t have (red).

Under CLiP/OCPP each laboratory was equipped with items versatile enough to allow for the extraction, isolation, identification and quantification of microplastics in water, sediments and biota. Laboratory equipment provided was portable and modulable allowing for easy storage when not in use to preserve laboratory space and to allow for easy scale-up when required. This modulable and scalable aspect particularly applied to such items as mini shaker incubators required for sample incubation as well as vacuum manifolds for sample filtration. While the laboratory items represented a substantial once-off investment (about £25k per laboratory excluding the ATR-FTIR) the total spend was still considered to be cost-effective. An independent audit carried out by itad in the UK (specialists in monitoring, evaluation and learning) concluded that the creation of those type of facilities was cost-effective when considering the subsequent outputs^48^. Such outputs included the creation of facilities capable of supporting diverse research projects including long-term monitoring programmes and amplifying in country capacity through training of experts in the field of marine litter research.

Each laboratory was provided with an ATR-FTIR (either a Thermo Scientific Nicolet iS5, Agilent Cary 630 FTIR Spectrometer or a Bruker ALPHA-II infrared spectrometers with polymer libraries) to allow for the identification of particles down to ~ 300 μm. Particle characterisation using spectroscopic techniques such as FTIR is the minimum requirement to produce robust data sets on microplastics. Particle characterisation and validation according to polymer type is essential for the accurate reporting of plastic items with the differentiation of natural items otherwise reported as plastics in many cases. Other techniques, such as the ‘hot needle test’, while cost-effective, is no longer accepted in current efforts to increase validity and to abide by minimum quality control criteria. To ensure accurate reporting and prevent misidentification of potential microplastics, it is imperative to utilise dependable and standardised techniques for characterizing and validating microplastic particles. Polymer identification techniques are often costly, requiring a level of training and expertise in order to accurately identify and characterise microplastic samples (Tables 1 and 3).

**Table 3:** Training matrix for microplastics analysis using the Nile red method. Level of skill needed:

		Low	Medium	High
	Survey design		✔	✔
Sampling	Site selection		✔	✔
	Deployment—Manual grab sampler		✔	
	Deployment—Manta trawl		✔	
	Sample collection—biota		✔	
	Sample collection—water	✔		
	Sample collection—riverine sediment	✔	✔	
Laboratory processes: Sediment	Sediment drying	✔		
	Density separation with SMI units	✔	✔	
	Chemical digestion		✔	
	Filtration	✔		
	NR staining	✔		
Laboratory processes: Biota	Dissection		✔	
	Chemical digestion		✔	
	Filtration	✔		
	NR staining	✔		
Laboratory processes: Water	Chemical digestion		✔	
	Filtration	✔		
	NR staining	✔		
Particle characterisation	Visualisation—microscopy		✔	
	Single particle picking		✔	
	Polymer identification using ATR-FTIR		✔	
	Polymer identification using μ-FTIR			✔
Quantification and reporting	Quantification		✔	
	Blank correction		✔	
	Reporting		✔	
	Statistical analysis		✔	✔
	Spatial mapping		✔	✔

Compromises had to be made to ensure the different laboratories were equipped with polymer identification tools while keeping operating costs low. Smaller sized items can however be analysed depending on their morphology including fibres when optimising the surface in contact with the ATR crystal. The size range supported using ATR-FTIR is especially suitable for surface water samples typically collected using 300 μm mesh size nets. The limitation would therefore be for seafloor sediment samples and biota for which particles captured tend to be smaller in size. Between 1 and 10% of particles from seafloor sediments, riverine sediments and biota have previously been identified using ATR-FTIR^29–32^.

## The need for in country staff building capacity

Setting up appropriate infrastructure does require the training of competent lab-users. Capacity building was first achieved with Cefas staff visiting labs and training local staff on best practices for the collection of environmental samples as well as their processing and analysis in the laboratory. Best practices were mainly identified from national or international guidelines (e.g. OSPAR) for the sampling and processing of environmental samples for microplastics in the form of SOPs. A number of SOPs were previously developed and tested at Cefas and UEA and consisted of SOPs on sample and laboratory protocols. Training within the MyMiP network involved UK-based training for the network coordinator, followed by in-country training and provision of SOPs and training videos (English and Malay).

All SOPs were optimised with country partners to allow for country specific requirements, challenges and to take into account extremely valuable local expertise especially in terms of sampling locations, land uses, main likely sources of plastic contamination and biota abundance and distribution.

A training matrix was developed and is presented in Table 3. Most analytical steps required a skill level of low to medium (Fig. 4). This particularly applied to analytical steps such as drying of the sediment, density extraction and chemical digestion. Higher levels of skills were however required for specific steps including analysis of Nile Red staining results, polymer identification using μ-FTIR, statistical analysis of data and spatial mapping using GIS applications.

## The need for reproducibility

Currently, microplastic research is not only facing several challenges such as the lack of reproducibility between laboratories but also between studies. The consequence of incomparable data is that projects and studies may only serve as “snapshots in time” and it will be challenging to report data further than individual studies, impeding the international objectives under Regional Seas Conventions, or indeed the UN Sustainable Development Goals. One solution is the common adoption of standardised protocols and harmonised guidelines for the sampling, processing and reporting of microplastics in environmental samples. The notion of harmonisation has been described by the EUROqCHARM project (www.EUROqCHARM.eu) as the development of a cluster of monitoring procedures including—sampling strategy, sample collection, handling and storage, sample preparation, analysis, quality assurance and control criteria, data management protocols—that provide cross-comparable data and are validated using certified reference material (CRM)^49^. By breaking the analytical pathway down into useable elements, researchers can focus on optimising and testing the reproducibility of each step. It also allows updates in monitoring guidelines based on method elements, rather than revisions of full guidelines^9^. Strategic survey design and sampling approach is a fundamental element of monitoring that is often overlooked, although there are several internationally recommended sampling methods across a variety of matrices. The differences between the recommended sample methods are being explored. The situation is further compounded when processing and analysing samples in the laboratory, given the many different combinations of approaches available each with strengths and limitations. Current projects are focusing on developing interlaboratory proficiency tests on microplastics including QUASIMEME supported by the NORMAN network and more recently by the international project EUROqCHARM^45^. International efforts to align analytical protocols for microplastics analysis are listed in Table 4. To date, no interlaboratory proficiency test focussed entirely on the use of NR coupled with ATR-FTIR for the analysis of microplastics in environmental samples. Many of the microplastic interlaboratory comparison studies (ILCs) to date have been hampered by the diversity of methods applied by each participant. Therefore, it is paramount that an ILC is performed where there are no significant modifications in methodology which hamper comparative data analysis.

**Table 4:** List of past and current method development exercises and proficiency tests for the analysis of microplastics in simple and complex matrices including current work being developed under OCPP.

Description	Organisers	Round number	Year	Particle type	Targeted matrices	Link/reference to report/output	Link to website
An interlaboratory comparison exercise for the determination of microplastics in standard sample bottles	Ministry of the Environment, Japan	–	2019	Microplastics (0.4–5.7 mm)	Seawater	^50^	
Microplastics analysis development exercise	Quasimeme/NORMAN	1	2019	-Pre-production pellets -Microplastics	-Tablets with plastic particles	^45^	https://www.wepal.nl/en/wepal/Home/Proficiency-tests.htm
Microplastics analysis development exercise	Quasimeme/NORMAN	2	2020	Microplastics	-Tablets with plastic particles -Fish -Sediment	Report circulated	https://www.wepal.nl/en/wepal/Home/Proficiency-tests.htm
Microplastics analysis development exercise	Quasimeme/NORMAN in collaboration with the EU project EUROqCHARM	3	2022	Microplastics	Water, sediment	–	https://www.euroqcharm.eu www.quasimeme.org)
Proficiency test	JRC/BAM	–	2020	Microplastics	Water	Outputs of the exercise will be covered during a symposium: “Challenges of microplastic analysis—Bridging state of the art and policy needs” in September 2021	https://ec.europa.eu/jrc/en/news/finding-right-methods-measuring-microplastics-water
Project Missouri	–	–	–	–	Soil	Report circulated	https://www.ineris.fr/en/ineris/news/microplastics-ineris-leads-european-missouri-project
Norman Network	Joint Programme of Activities 2023	–	2023	WG-4 Sandwich Filters for the Comparison of Microplastic Measurements	Filters	–	WELCOME TO THE NORMAN NETWORK | NORMAN (norman-network.net)
Interlaboratory proficiency test on the use of Nile red for microplastic reporting	Cefas/UEA with support from EUROqCHARM	1	Planned for 2024	-Pre-production pellets -Microplastics	Water	Tablets with plastic particles	N/A

In bold: Planned activities under OCPP.

One of the tasks under the OCPP is the delivery of a first of its kind interlaboratory proficiency test on the analysis and reporting of microplastics above 300 μm (corresponding to the main mesh size used for surface water particles using manta or plankton nets) in clean water samples. All the laboratories (n = 15) expressed their interest in participating in this interlaboratory exercise highlighting the need from researchers and governmental bodies to produce reproducible data. Proficiency in the use of ATR-FTIR will be assessed using pre-production plastic pellets of various polymer composition. The first exercise of the WEPAL-QUASIMEME/NORMAN interlaboratory method development reported high variations between laboratories in the case of the pre-production plastic pellets suggesting the need for additional training in some cases^45^.

## Current use of the laboratories

A questionnaire was circulated between the project partners (n = 15) to understand current and planned use of the overseas laboratories. Additional information on methodology as well as a copy of the questionnaire can be found in Supplementary files.

The objective was to investigate whether the laboratories were currently active and determine any scientific needs prioritised for the future to understand what is feasible with the current setup and the knowledge or infrastructure gaps for any planned work. A copy of the questionnaire is provided in the SI Sections 14 laboratories out of 15 are currently using the facilities provided. One of the facilities was not currently in use due to a lack of human resources with the need of specialised technicians for laboratory management. Recent updates confirmed investment in staff with the identification of a specialised technician for the laboratory management.

Project partners were asked about the most urgent research topics, scientific questions or monitoring programmes which they would like to develop. The responses were divided into those relating to research and development (method developments, baselines, understanding impacts, understanding sources, pathways, transport and fate) and also those relating to monitoring, and are summarised in Table 5. It is important to understand this, as the use of the laboratories and priorities will define the methods set out, the information also offers opportunity to set up centralised resources and networks supporting scientific advances for these topics. The responses show that priorities are similar across the world, and across labs. Understanding baselines (n = 10) and understanding sources, pathways, transport and fate (n = 10) were most common responses. Priorities relating to monitoring activities came from both government (n = 6) and university responses (n = 2), these can be distinguished into different questions which drive monitoring, as described by Hutto & Belote (2013) with most referring to surveillance monitoring (n = 7), and just one response (Vanuatu government) interested in effectiveness monitoring to measure the success of their recent policy including a ban on single use items^51^ .

**Table 5:** Current use of the laboratories and priorities per institution (n = 15).

Continent	Country	Institution	Institution type	Current use and priorities					Comments
				Method development	Baseline	Understanding impacts	Understanding sources, pathways, transport, and fate	Monitoring	
Africa									
	South Africa	DFFE	Governmental	✔		✔			
	South Africa	SAEON	Governmental		✔		✔		
America									
	Belize	DoE	Government	✔	✔	✔	✔	✔	
	Belize	UB	University		✔	✔	✔	✔	
Australia									
	Vanuatu	Bureau of Standards	Government					✔	Effectiveness monitoring
Asia									
	Indonesia	BRIN	Government	✔	✔	✔	✔	✔	
	Maldives	MMRI	Government		✔	✔			
	Malaysia	Universitiy Kebangsaan	University				✔		
	Malaysia		University				✔		
	Philippines		University	✔	✔	✔			
	Sri Lanka	ITI	Government		✔		✔		
	Sri Lanka	NARA	Government			✔		✔	
	Sri Lanka	MEPA	Government			✔	✔	✔	
	Sri Lanka	CEA	Government		✔		✔		
Europe									
	United Kingdom	Cefas	Government	✔	✔		✔	✔	
	United Kingdom	UEA	University	✔	✔		✔	✔	

## Challenges and lessons learned

Some challenges occurred during the installation of the overseas laboratories. While some challenges were shared between all countries, additional obstacles were faced by some countries depending on individual national regulation and processes. Some of the lessons learned are listed below:

(i) Ensure use of the same terminology
(ii) Optimisation of protocols with in-country partners through R&D to address local challenges but keeping a global harmonisation of the outputs.
(iii) Anticipate delays from suppliers and look for alternatives when needed.
(iv) Anticipate storage issues for delivery of equipment.
(v) Use local suppliers as much as possible for chemicals and large equipment.
(vi) Work alongside in-country partners to avoid interfering with their usual daily activities and deliverables.
(vii) In-country visits are essential for effective lab installation and staff training.

Shipping logistics and customs processes vary greatly across countries and should also be investigated early in the process to avoid delays in shipment releases and delivery at destination. Use of international shipping agents allowed for a smoother process covering item pickup from the point of origin to shipping, customs release and delivery at destination. It was very important to cover most fees regarding customs and transport fees to avoid putting additional financial strains on the project partners. Li-ion batteries create additional issues for air freight in particular and should be avoided or procured locally where possible to avoid additional expense.

## Current work and next steps

**Figure 5:**
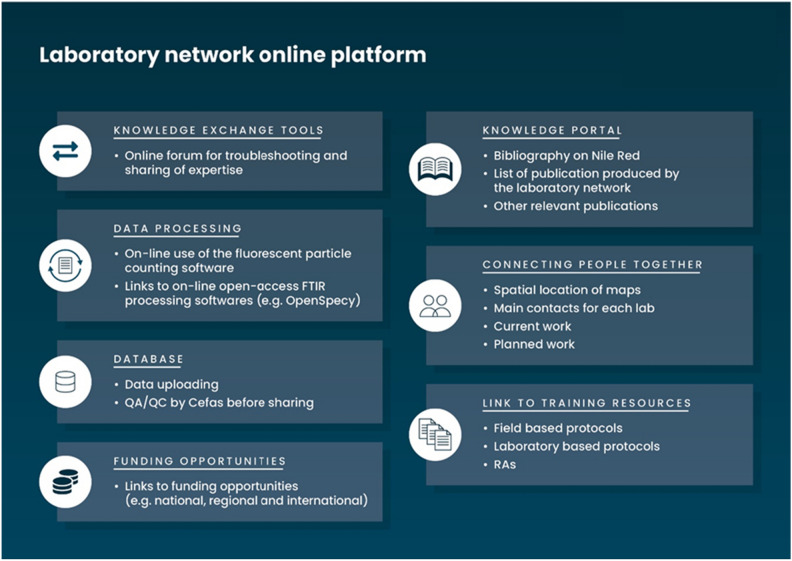
Planned content of the laboratory network on-line platform.

Current work is being carried out to ensure a sustainable engagement and connectivity amongst, otherwise, isolated national microplastics laboratories facilities. It was a long-term vision during the first stage of the project (CLiP) to create a common shared on-line platform to ensure dialogue between the different stakeholders as well as developing tools to allow knowledge sharing. Online resources are currently being developed under OCPP to ensure that local expertise is being shared and that local or regional issues could be resolved by learning from best practices and lessons learned in other countries. Some features of the online platform will include (Fig. 5):

(i) Connecting people together: Spatial map of the locations of the laboratories with key contacts and main work being carried out.
(ii) Knowledge transfer hub: Linking together users and appropriate training materials for both sample collection in the field and analysis in the laboratory. The knowledge transfer hub will also contain relevant bibliography (i.e. use of NR) as well as publications and reports produced by the laboratory network.
(iii) Facilitating data processing: An automatic particle counting tool based on fluorescence detection will be embedded and made available use on-line. This will assist with consistency in practice and data interpretation.
(iv) Data submission and management: A database will also be available for short to longer term data storage. Microplastics data will be submitted to Cefas for QA/QC analysis of the data and will be uploaded accordingly. The proposed database will be aligned with current widely used databases for microlitter (including microplastics) (i.e. ICES DOME or EMODnet chemistry) but will be simplified in terms of vocabulary and mandatory reporting fields.
(v) Sustainable hub: Funding opportunities (e.g. national, regional or international) will be shared between users to allow for longer term self-sustainable use of the laboratories.

### Supplementary Information

Supplementary Information.

## Data Availability

Not applicable.

## Abbreviations

ATR-FTIR: Attenuated Total Reflectance Fourier Transform Infrared Spectroscopy
BPF: Blue Planet Fund
CCOA: Commonwealth Clean Ocean Alliance
CEA: Central Environment Authority—Sri Lanka
Cefas: Centre for Environment, Fisheries and Aquaculture Science—UK
CLiP: Commonwealth Litter Programme
CRMs: Certified Reference Materials
DEFF: Department of Agriculture, Forestry and Fisheries—South Africa
Defra: Department for Environment, Food and Rural Affairs—UK
DoE: Department of the Environment—Belize
FOA: Friends of Ocean Action
GC-MS: Gas chromatography–mass spectrometry
GOAP: Global Ocean Accounts Partnership
GoB: Government of Belize
IAEA: International Atomic Energy Agency
ITI: Industrial Technology Institute—Sri Lanka
JNCC: Joint Nature Conservation Committee—UK
KOH: Potassium hydroxide
LIPI: Indonesian Institute of Sciences
MEPA: Marine Environment Protection Authority—Sri Lanka
μ-FTIR: Micro Fourier Transform Infrared Spectroscopy
MLAP: Marine Litter Action Plan
MMO: Marine Management Organisation—UK
MMRI: Maldives Marine Research Institute
MSDS: Material Safety Data Sheet
NARA: National Aquatic Resources Research and Development Agency—Sri Lanka
NaClO: Sodium hypochlorite
NC filters: Cellulose nitrate filters
NR: Nile red
NUTEC: NUclear TEChnology for Controlling Plastic Pollution (NUTEC Plastics)
OCPP: Ocean Country Partnership Programme
ODA: Official Development Assistance
OHA: One Health Aquaculture
RC filters: Regenerated cellulose filters
RCO BRIN: Research Center for Oceanography National Research and Innovation Agency of the Republic of Indonesia
RM: Reference materials
RO: Reverse Osmosis
SA: South Africa
SAEON: South African Environmental Observation Network
SMI: Sediment—Microplastic Isolation unit SOPs: Standard Operating Procedures
UB: University of Belize
UEA: University of East Anglia—UK

## References

[1] Gigault J (2018). Current opinion: What is a nanoplastic?. Environ. Pollut..

[2] GESAMP. Guidelines for the monitoring and assessment of plastic litter in the ocean, GESAMP (2019).

[3] Miller ME, Hamann M, Kroon FJ (2020). Bioaccumulation and biomagnification of microplastics in marine organisms: A review and meta-analysis of current data. PLoS One.

[4] Walker, D. et al. A critical review of microbiological colonisation of nano- and microplastics (NMPs) and their significance to the food chain (2022).

[5] Vianello A, Jensen RL, Liu L, Vollertsen J (2019). Simulating human exposure to indoor airborne microplastics using a Breathing Thermal Manikin. Sci. Rep..

[6] Cox KD, Covernton GA, Davies HL, Dower JF, Juanes F, Dudas SE (2019). Human consumption of microplastics. Environ. Sci. Technol..

[7] Blackburn K, Green D (2022). The potential effects of microplastics on human health: What is known and what is unknown. Ambio.

[8] Rochman CM (2019). Rethinking microplastics as a diverse contaminant suite. Environ. Toxicol. Chem..

[9] Aliani S (2023). Reproducible pipelines and readiness levels in plastic monitoring. Nat. Rev. Earth Environ..

[10] Kohler, P. et al. A blue future: Developing a national marine litter action plan in SIDS—lessons learnt from a successful story in Belize. *Prep, This Issue* (2021).

[11] Cefas, Commonwealth Litter Programme (2020). https://www.cefas.co.uk/clip/about-clip/. Accessed 11 Feb 2023.

[12] Shahul Hamid F, Bhatti MS, Anuar N, Anuar N, Mohan P, Periathamby A (2018). “Worldwide distribution and abundance of microplastic: How dire is the situation?. Waste Manag. Res..

[13] Yusuf A (2022). Updated review on microplastics in water, their occurrence, detection, measurement, environmental pollution, and the need for regulatory standards. Environ. Pollut..

[14] Bakir A (2023). A spatial and temporal assessment of microplastics in seafloor sediments: A case study for the UK. Front. Mar. Sci..

[15] Nel HA, Naidoo T, Akindele EO, Nhiwatiwa T, Fadare OO, Krause S (2021). Collaboration and infrastructure is needed to develop an African perspective on micro (nano) plastic pollution. Environ. Res. Lett..

[16] Maes T, Jessop R, Wellner N, Haupt K, Mayes AG (2017). A rapid-screening approach to detect and quantify microplastics based on fluorescent tagging with Nile Red. Sci. Rep..

[17] Meyers N (2022). Microplastic detection and identification by Nile red staining: Towards a semi-automated, cost-and time-effective technique. Sci. Total Environ..

[18] Shruti VC, Pérez-Guevara F, Roy PD, Kutralam-Muniasamy G (2021). Analyzing microplastics with Nile Red: Emerging trends, challenges, and prospects. J. Hazard. Mater..

[19] Ho, D., Liu, S., Wei, H., & Karthikeyan, K. G. The glowing potential of Nile red for microplastics Identification: Science and mechanism of fluorescence staining. *Microchem. J.* 109708 (2023).

[20] Wang C, Jiang L, Liu R, He M, Cui X, Wang C (2021). Comprehensive assessment of factors influencing Nile red staining: Eliciting solutions for efficient microplastics analysis. Mar. Pollut. Bull..

[21] Erni-Cassola G, Gibson MI, Thompson RC, Christie-Oleza JA (2017). Lost, but found with Nile Red: A novel method for detecting and quantifying small microplastics (1 mm to 20 μm) in environmental samples. Environ. Sci. Technol..

[22] Nalbone L, Panebianco A, Giarratana F, Russell M (2021). Nile Red staining for detecting microplastics in biota: Preliminary evidence. Mar. Pollut. Bull..

[23] Nel HA (2021). Detection limits are central to improve reporting standards when using Nile red for microplastic quantification. Chemosphere.

[24] Prata JC, Reis V, Matos JTV, da Costa JP, Duarte AC, Rocha-Santos T (2019). A new approach for routine quantification of microplastics using Nile Red and automated software (MP-VAT). Sci. Total Environ..

[25] Kukkola A (2023). Easy and accessible way to calibrate a fluorescence microscope and to create a microplastic identification key. MethodsX.

[26] Kukkola AT (2022). A large-scale study of microplastic abundance in sediment cores from the UK continental shelf and slope. Mar. Pollut. Bull..

[27] Wang Z (2018). Preferential accumulation of small (< 300 μm) microplastics in the sediments of a coastal plain river network in eastern China. Water Res..

[28] Catarino AI, Macchia V, Sanderson WG, Thompson RC, Henry TB (2018). Low levels of microplastics (MP) in wild mussels indicate that MP ingestion by humans is minimal compared to exposure via household fibres fallout during a meal. Environ. Pollut..

[29] Bakir A (2020). Microplastics in commercially important small pelagic fish species from South Africa. Front. Mar. Sci..

[30] Silburn, B. et al. A baseline study of macro, meso and micro litter in the Belize River basin, from catchment to coast. ICES J. Mar. Sci. (2022).

[31] Bakir A (2020). Occurrence and abundance of meso and microplastics in sediment, surface waters, and marine biota from the South Pacific region. Mar. Pollut. Bull..

[32] Preston-Whyte F (2021). Meso-and microplastics monitoring in harbour environments: A case study for the Port of Durban, South Africa. Mar. Pollut. Bull..

[33] Margenat H, Nel HA, Stonedahl SH, Krause S, Sabater F, Drummond JD (2021). Hydrologic controls on the accumulation of different sized microplastics in the streambed sediments downstream of a wastewater treatment plant (Catalonia, Spain). Environ. Res. Lett..

[34] Kukkola, A. et al. Prevailing impacts of river management on microplastic transport in contrasting US streams: Rethinking global microplastic flux estimations. *Water Res.* 120112 (2023). 10.1016/j.watres.2023.120112 37257293

[35] Kelleher L (2023). Microplastic accumulation in endorheic river basins–The example of the Okavango Panhandle (Botswana). Sci. Total Environ..

[36] ThermoFisher Scientific, Analytical Instruments for Microplastics Analysis (2020).

[37] Bakir, A., Russell, J., & Maes, T. owards a protocol for the observation of microplastics in Biota WP 5.3: Indicator for ingestion of microplastics (2020). [Online]. https://www.cleanatlantic.eu/wp-content/uploads/2021/05/5.3-Towards-a-protocol-for-observation-of-microplastic-in-biota.-Final_Cefas.pdf.

[38] Lusher AL, Primpke S (2023). Finding the balance between research and monitoring: When are methods good enough to understand plastic pollution?. Environ. Sci. Technol..

[39] Bessa et al. Harmonized protocol for monitoring microplastics in biota (2019).

[40] Gago et al. Standardised protocol for monitoring microplastics in seawater (2018).

[41] Frias, J. Standardised protocol for monitoring microplastics in sediments (2018).

[42] AMAP, Litter and microplastics monitoring guidelines. Version 1.0. Tromsø, Norway (2021).

[43] HELCOM, HELCOM Guidelines on monitoring of microlitter in seabed sediments in the Baltic Sea (2022).

[44] Galgani, F. et al., Guidance on monitoring of marine litter in European Seas—Update of the guidance on monitoring of marine litter for the Marine Strategy Framework Directive. Luxembourg (2023).

[45] Van Mourik LM (2021). Results of WEPAL-QUASIMEME/NORMANs first global interlaboratory study on microplastics reveal urgent need for harmonization. Sci. Total Environ..

[46] Aminah IS, Ikejima K (2023). Potential sources of microplastic contamination in laboratory analysis and a protocol for minimising contamination. Environ. Monit. Assess..

[47] Pfeiffer F, Fischer EK (2020). Various digestion protocols within microplastic sample processing—Evaluating the resistance of different synthetic polymers and the efficiency of biogenic organic matter destruction. Front. Environ. Sci..

[48] itad, Cefas CLiP Project Value for Money Assessment (2021).

[49] EUROqCHARM, EUROqCHARM (2020).

[50] Isobe A (2019). An interlaboratory comparison exercise for the determination of microplastics in standard sample bottles. Mar. Pollut. Bull..

[51] Department of Environmental Protection and Conservation, Plastic Ban (2023). https://environment.gov.vu/index.php/environmental-protection/plastic-ban.

