# Supplementary material for: Creation of an international laboratory network towards global microplastics monitoring harmonisation
Source: Sci Rep. 2024 Jun 3;14:12714. doi: 10.1038/s41598-024-62176-y (PMC11148021; doi:10.1038/s41598-024-62176-y)
Supplement: Supplementary file 1 — Supplementary Information. [file 41598_2024_62176_MOESM1_ESM.docx]

**Supplementary files**

**Current use of the laboratories**

1. **Introduction**

This section presents the approach taken to gather information on the current uses of the laboratory facilities as well as current priorities. The main aim was to circulate a questionnaire to collect evidence on laboratory use as well as types of activities were being carried out for each country according to research and policy priorities. The main objectives were: i) to gather information on scientific priorities per country, ii) to investigate laboratory use, iii) to gather information on current research of monitoring programmes being conducted as well as the target compartment (i.e. beach sand, seafloor sediment, surface water, water column, biota, marine or freshwater), iv) to identify issues related to sample analysis related to matrix, v) to gather information on future/planned activities, vi) to highlight any missing equipment or training gaps and vii) to gather information on the willingness for partners to be involved in an interlaboratory proficiency test on the use of Nile red coupled with FTIR for microplastics analysis.

1. **Materials and methods**

All partners were invited to complete a questionnaire consisting of 11 questions. All questions including the invitation letter can be found below is provided in Appendix I. Questions were designed to support the main aim and objectives listed previously.

Appendix I. Invitation letter circulated to country partners as well as questions asked.

**‘OCPP Laboratory Network**

Dear project partner, we would like to invite you to co-author a publication entitled ‘**Creation of an international laboratory network – A step towards a global harmonisation for microplastics monitoring**’. This publication will focus on how to create a microplastic laboratory in the context of the Commonwealth Litter Programme (CLiP) and now the Ocean Country Partnership Programme (OCPP).

We would like to ask you for your input which will be the central focus of the publication. Would you mind answering a few questions below? Your replies (with your approval) will be combined in a table which will be added in the supplementary information section. The replies will be kept anonymous. The questions are as follows:

1. What are your most urgent research topics/scientific questions or monitoring programmes you would like to develop.
2. Are you currently using the microplastic laboratory that was supported under either CLiP or OCPP: **Yes or No**
3. If No, please specify the main reasons why.
4. If yes, please specify the main reason it is still in use.
5. If yes, please describe the research or monitoring programme being conducted.
6. Please specify the main environmental compartment(s) being investigated (i.e. beach sand, seafloor sediment, surface water, water column, biota, marine or freshwater).
7. According to your processes, which environmental compartment (i.e. matrix) is the less problematic to work with (e.g. water) and the most problematic (e.g. sediment) ?
8. Please provide the names of any publications/reports produced using the microplastics laboratories.
9. What are your future plans, visions for the laboratory (e.g. expand the scope of the research including water quality measurements, food safety, etc….).
10. According to your experience, what key components (i.e. equipment, training or specific skills) are currently missing in your microplastic laboratory.
11. Would you be interested in being involved in the first of its kind interlaboratory method development exercise based on Nile red and ATR-FTIR analysis of particles above 300 μm in size. ‘
12. **Results and discussion**

Responses from the different laboratories (n=15) were processed in excel and responses logged per institution. Results were discussed in the main text in section 6.
